# Dichloridobis[(ferrocenyl­methyl­idene)(furan-2-ylmeth­yl)amine-κ*N*]palladium(II)

**DOI:** 10.1107/S1600536812009191

**Published:** 2012-03-07

**Authors:** William M. Motswainyana, Martin O. Onani, Roger A. Lalancette

**Affiliations:** a Chemistry Department, University of the Western Cape, Modderdam Road, Private bag X17, Bellville, 7535, South Africa; b Carl A. Olson Memorial Laboratories, Department of Chemistry, Rutgers University, Newark, NJ 07102 USA

## Abstract

The title compound, [Fe_2_Pd(C_5_H_5_)_2_(C_11_H_10_NO)_2_Cl_2_], exhibits a square-planar geometry at the Pd^II^ atom, which is determined by inversion-related chlorine and ferrocenyl­imine mol­ecules across a center of symmetry. The ferrocenyl­imine moieties are *trans* to each other.

## Related literature

For the synthesis of ferrocenyl­imine ligands and their transition metal-based complexes, see: Mu *et al.* (2007 ▶); Lu *et al.* (2007 ▶); Pou *et al.* (2007 ▶); Neuse *et al.* (1988 ▶). For related structures, see: Rajput *et al.* (2004 ▶, 2006 ▶); Nelana *et al.* (2008 ▶). For related applications, see: Stang *et al.* (1996 ▶); Pou *et al.* (2007 ▶). For Pd—Cl bond lengths, see: Allen (2002 ▶). For the preparation of the precursor mol­ecule, see: Salo & Guan (2003 ▶).
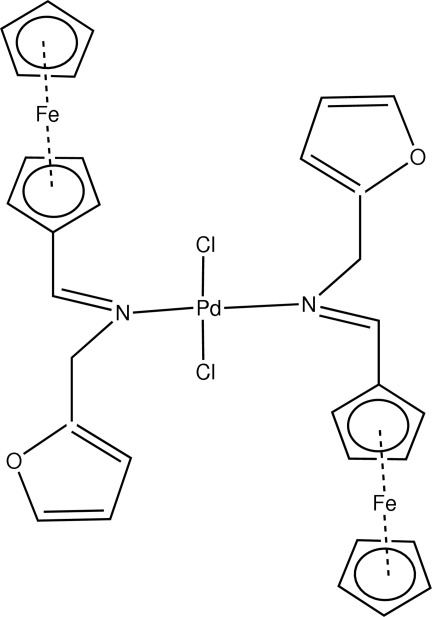


## Experimental

### 

#### Crystal data

[Fe_2_Pd(C_5_H_5_)_2_(C_11_H_10_NO)_2_Cl_2_]
*M* *_r_* = 763.58
Monoclinic, 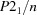

*a* = 12.2113 (7) Å
*b* = 7.3439 (5) Å
*c* = 16.365 (1) Å
β = 100.616 (4)°
*V* = 1442.44 (16) Å^3^
*Z* = 2
Cu *K*α radiation
μ = 14.91 mm^−1^
*T* = 100 K
0.44 × 0.07 × 0.04 mm

#### Data collection

Bruker SMART CCD APEXII area-detector diffractometer
Absorption correction: numerical (*SADABS*; Sheldrick, 2008*a* ▶; Parkin *et al.*, 1995 ▶) *T*_min_ = 0.059, *T*_max_ = 0.560
12086 measured reflections
2598 independent reflections
1886 reflections with *I* > 2σ(*I*)
*R*_int_ = 0.088

#### Refinement

*R*[*F* ^2^ > 2σ(*F* ^2^)] = 0.043
*wR*(*F* ^2^) = 0.109
*S* = 1.02
2598 reflections
187 parameters
H-atom parameters constrained
Δρ_max_ = 0.85 e Å^−3^
Δρ_min_ = −1.01 e Å^−3^

### 

Data collection: *APEX2* (Bruker, 2006 ▶); cell refinement: *SAINT* (Bruker, 2005 ▶); data reduction: *SAINT*; program(s) used to solve structure: *SHELXTL* (Sheldrick, 2008*b* ▶); program(s) used to refine structure: *SHELXTL*; molecular graphics: *SHELXTL*; software used to prepare material for publication: *SHELXTL*.

## Supplementary Material

Crystal structure: contains datablock(s) I, New_Global_Publ_Block, global. DOI: 10.1107/S1600536812009191/pk2391sup1.cif

Structure factors: contains datablock(s) I. DOI: 10.1107/S1600536812009191/pk2391Isup2.hkl

Additional supplementary materials:  crystallographic information; 3D view; checkCIF report

## Figures and Tables

**Fig. 1.**
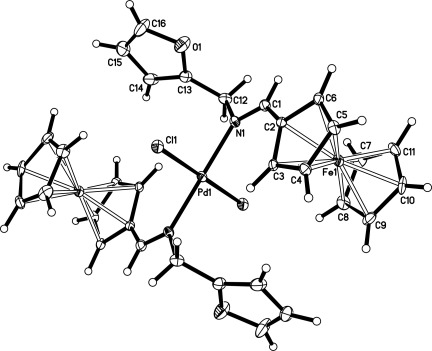
A view of the molecular structure with displacement ellipsoids drawn at the 30% probability level for non-H atoms.

## supplementary crystallographic information

### Comment

Ferrocenyl derivatives containing good donor atoms have evoked research interest because their coordination to another metal produces multicentered molecules where the two metals are in close proximity but in different environments. This property may influence the mutual cooperation of the metals in a variety of application processes (Stang *et al.*, 1996; Rajput *et al.*, 2004; Rajput *et al.*, 2006; Neuse *et al.*, 1988; Pou *et al.*, 2007). For instance, some ferrocenyl complexes have displayed promising cytotoxicity profiles (Neuse *et al.*, 1988; Pou *et al.*, 2007). Preference for these complexes is derived from their convenience of preparation, facile modification and handling (Mu *et al.*, 2007; Lu *et al.*, 2007). In an attempt to prepare new bulky bis(ferrocenylimine) palladium^II^ complexes which could induce apoptosis on tumor cells, the title compound was obtained.

The molecular structure contains one molecule of the Pd^II^ complex (Fig. 1) across a center of symmetry (one-half of the molecule is the asymmetric unit). All bond lengths and angles are normal and comparable with those reported for similar structures (Rajput *et al.*, 2004; Nelana *et al.*, 2008; Pou *et al.*, 2007). The Pd^II^ ion has square planar coordination geometry around the metal center coordinated to two ferrocenylimine ligands *via* the imine nitrogen atoms and the chloride ions. The ferrocenylimine molecules are *trans* to each other across the center of symmetry. There is no *trans* influence observed for the chloride ligands: the Pd–Cl bond length is in agreement with known Pd–Cl bond distances for palladium complexes (Allen, 2002).

### Experimental

[PdCl_2_(cod)] was prepared following literature method (Salo & Guan, 2003). To a suspension of PdCl_2_(cod) [0.0394 g, 0.138 mmol] in a mixture of CH_2_Cl_2_/Et_2_O (20 ml) was added a solution of ferrocenyl-2-furylmethyl)imine (0.0801 g, 0.2732 mmol) in CH_2_Cl_2_ (5 ml). An orange precipitate was observed immediately. The reaction was stirred at room temperature for 12 hrs. The precipitate was filtered off, washed with dry hexane (2 *x* 5 ml) and dried under vacuum to yield an orange solid. Recrystallization of the product was done from a mixture of CH_2_Cl_2_:*C*_6_H_14_ which gave single crystals which were used for the X-ray diffraction studies. The yield of the product was 0.0738 g which translates to 70%.

### Refinement

All H atoms for (I) were found in electron density difference maps. The methylene, methine, furanyl & cyclopentadienyl Hs were placed in geometrically idealized positions and constrained to ride on their parent C atoms with C—H distances of 0.99, 1.00, 0.95, and 0.95 Å, respectively, and *U*_iso_(H) = 1.2*U*_eq_(C). The low fraction of data collected may affect the precision of the structure.

An additional empirical absorption correction was made using the program XABS2 (Parkin *et al.*, 1995), which flattened the residual difference map features from 1.60 and -1.51 eÅ^-3^ to 0.85 and -0.10 eÅ^-3^ and lowered *R*_1_ to 4.30% from 5.50%.

### Crystal data

**Table:**

[Fe_2_Pd(C_5_H_5_)_2_(C_11_H_10_NO)_2_Cl_2_]	*F*(000) = 768
*M**_r_* = 763.58	*D*_x_ = 1.758 Mg m^−^^3^
Monoclinic, *P*2_1_/*n*	Cu *K*α radiation, λ = 1.54178 Å
Hall symbol: -P 2yn	Cell parameters from 5083 reflections
*a* = 12.2113 (7) Å	θ = 4.2–71.6°
*b* = 7.3439 (5) Å	µ = 14.91 mm^−^^1^
*c* = 16.365 (1) Å	*T* = 100 K
β = 100.616 (4)°	Needle, red
*V* = 1442.44 (16) Å^3^	0.44 × 0.07 × 0.04 mm
*Z* = 2	

### Data collection

**Table:**

Bruker SMART CCD APEXII area-detector diffractometer	2598 independent reflections
Radiation source: fine-focus sealed tube	1886 reflections with *I* > 2σ(*I*)
Graphite monochromator	*R*_int_ = 0.088
φ and ω scans	θ_max_ = 71.6°, θ_min_ = 4.2°
Absorption correction: numerical (*SADABS*; Sheldrick, 2008*a*; Parkin *et al.*, 1995)	*h* = −14→14
*T*_min_ = 0.059, *T*_max_ = 0.560	*k* = −7→8
12086 measured reflections	*l* = −18→19

### Refinement

**Table:**

Refinement on *F*^2^	Primary atom site location: structure-invariant direct methods
Least-squares matrix: full	Secondary atom site location: difference Fourier map
*R*[*F*^2^ > 2σ(*F*^2^)] = 0.043	Hydrogen site location: inferred from neighbouring sites
*wR*(*F*^2^) = 0.109	H-atom parameters constrained
*S* = 1.02	*w* = 1/[σ^2^(*F*_o_^2^) + (0.053*P*)^2^] where *P* = (*F*_o_^2^ + 2*F*_c_^2^)/3
2598 reflections	(Δ/σ)_max_ < 0.001
187 parameters	Δρ_max_ = 0.85 e Å^−^^3^
0 restraints	Δρ_min_ = −1.01 e Å^−^^3^

### Special details

**Table:**

Experimental. 'Crystal mounted on a Cryoloop using Paratone-N.'
Geometry. All e.s.d.'s (except the e.s.d. in the dihedral angle between two l.s. planes) are estimated using the full covariance matrix. The cell e.s.d.'s are taken into account individually in the estimation of e.s.d.'s in distances, angles and torsion angles; correlations between e.s.d.'s in cell parameters are only used when they are defined by crystal symmetry. An approximate (isotropic) treatment of cell e.s.d.'s is used for estimating e.s.d.'s involving l.s. planes.
Refinement. Refinement of *F*^2^ against ALL reflections. The weighted *R*-factor *wR* and goodness of fit *S* are based on *F*^2^, conventional *R*-factors *R* are based on *F*, with *F* set to zero for negative *F*^2^. The threshold expression of *F*^2^ > σ(*F*^2^) is used only for calculating *R*-factors(gt) *etc*. and is not relevant to the choice of reflections for refinement. *R*-factors based on *F*^2^ are statistically about twice as large as those based on *F*, and *R*- factors based on ALL data will be even larger.

### Fractional atomic coordinates and isotropic or equivalent isotropic displacement parameters (Å^2^)

**Table:**

	*x*	*y*	*z*	*U*_iso_*/*U*_eq_	
Pd1	0.5000	0.0000	0.5000	0.01970 (17)	
Fe1	0.16463 (7)	0.24226 (12)	0.38773 (5)	0.0222 (2)	
Cl1	0.50057 (10)	−0.24917 (19)	0.41448 (8)	0.0259 (3)	
O1	0.6314 (3)	0.0915 (7)	0.2825 (3)	0.0447 (12)	
N1	0.4827 (3)	0.1692 (6)	0.4011 (3)	0.0216 (10)	
C1	0.3939 (4)	0.2003 (7)	0.3462 (3)	0.0237 (12)	
H1	0.4004	0.2900	0.3055	0.028*	
C2	0.2862 (4)	0.1145 (7)	0.3395 (3)	0.0212 (12)	
C3	0.2431 (4)	−0.0047 (8)	0.3950 (3)	0.0221 (11)	
H3	0.2866	−0.0615	0.4464	0.026*	
C4	0.1275 (4)	−0.0274 (8)	0.3641 (3)	0.0247 (13)	
H4	0.0752	−0.1019	0.3907	0.030*	
C5	0.0977 (5)	0.0775 (8)	0.2905 (3)	0.0243 (13)	
H5	0.0211	0.0886	0.2565	0.029*	
C6	0.1943 (4)	0.1658 (8)	0.2740 (3)	0.0243 (12)	
H6	0.1985	0.2483	0.2260	0.029*	
C7	0.2220 (5)	0.4917 (9)	0.4320 (4)	0.0366 (14)	
H7	0.2929	0.5502	0.4233	0.044*	
C8	0.2098 (5)	0.3762 (8)	0.4999 (3)	0.0333 (15)	
H8	0.2702	0.3391	0.5468	0.040*	
C9	0.0967 (5)	0.3196 (8)	0.4873 (4)	0.0327 (14)	
H9	0.0629	0.2367	0.5243	0.039*	
C10	0.0392 (5)	0.4038 (8)	0.4125 (4)	0.0330 (15)	
H10	−0.0417	0.3902	0.3882	0.040*	
C11	0.1169 (5)	0.5103 (9)	0.3793 (3)	0.0347 (14)	
H11	0.1008	0.5841	0.3271	0.042*	
C12	0.5830 (4)	0.2729 (8)	0.3932 (3)	0.0263 (13)	
H12A	0.6173	0.3222	0.4482	0.032*	
H12B	0.5617	0.3770	0.3551	0.032*	
C13	0.6655 (4)	0.1593 (8)	0.3610 (3)	0.0285 (13)	
C14	0.7705 (5)	0.1052 (10)	0.3911 (4)	0.0394 (16)	
H14	0.8138	0.1352	0.4437	0.047*	
C15	0.8039 (5)	−0.0051 (11)	0.3289 (4)	0.0503 (18)	
H15	0.8741	−0.0634	0.3322	0.060*	
C16	0.7198 (6)	−0.0120 (10)	0.2654 (5)	0.0526 (19)	
H16	0.7197	−0.0781	0.2155	0.063*	

### Atomic displacement parameters (Å^2^)

**Table:**

	*U*^11^	*U*^22^	*U*^33^	*U*^12^	*U*^13^	*U*^23^
Pd1	0.0167 (3)	0.0232 (3)	0.0167 (3)	0.0001 (2)	−0.00344 (19)	−0.0002 (2)
Fe1	0.0187 (4)	0.0246 (5)	0.0207 (5)	0.0013 (4)	−0.0032 (3)	−0.0016 (4)
Cl1	0.0254 (7)	0.0275 (8)	0.0216 (7)	0.0012 (6)	−0.0039 (5)	−0.0054 (6)
O1	0.022 (2)	0.071 (3)	0.038 (3)	0.004 (2)	−0.0021 (19)	−0.019 (2)
N1	0.019 (2)	0.022 (3)	0.023 (3)	−0.0030 (19)	−0.0002 (19)	0.0001 (19)
C1	0.026 (3)	0.026 (3)	0.019 (3)	0.002 (2)	0.003 (2)	−0.001 (2)
C2	0.021 (3)	0.023 (3)	0.018 (3)	0.001 (2)	0.001 (2)	−0.003 (2)
C3	0.019 (3)	0.026 (3)	0.019 (3)	0.006 (3)	−0.002 (2)	0.000 (3)
C4	0.019 (3)	0.028 (4)	0.025 (3)	−0.001 (2)	−0.002 (2)	−0.001 (2)
C5	0.022 (3)	0.024 (3)	0.023 (3)	0.003 (2)	−0.006 (2)	−0.007 (2)
C6	0.022 (3)	0.030 (3)	0.018 (3)	0.006 (2)	−0.004 (2)	−0.004 (2)
C7	0.039 (3)	0.031 (4)	0.039 (4)	−0.011 (3)	0.006 (3)	−0.018 (3)
C8	0.032 (3)	0.040 (4)	0.022 (3)	0.002 (3)	−0.010 (3)	−0.012 (3)
C9	0.035 (3)	0.032 (4)	0.035 (4)	0.003 (3)	0.015 (3)	−0.005 (3)
C10	0.023 (3)	0.029 (4)	0.043 (4)	0.009 (3)	−0.004 (3)	−0.010 (3)
C11	0.049 (4)	0.027 (3)	0.026 (3)	0.014 (3)	−0.002 (3)	0.003 (3)
C12	0.027 (3)	0.031 (4)	0.019 (3)	−0.005 (2)	0.001 (2)	−0.003 (2)
C13	0.019 (3)	0.040 (4)	0.026 (3)	−0.004 (3)	0.002 (2)	0.002 (3)
C14	0.023 (3)	0.066 (5)	0.027 (4)	−0.001 (3)	0.003 (3)	0.009 (3)
C15	0.027 (3)	0.074 (5)	0.052 (4)	0.011 (4)	0.012 (3)	0.017 (4)
C16	0.035 (4)	0.070 (5)	0.054 (4)	0.011 (4)	0.011 (3)	−0.026 (4)

### Geometric parameters (Å, º)

**Table:**

Pd1—N1	2.021 (4)	C4—C5	1.420 (7)
Pd1—N1^i^	2.021 (4)	C4—H4	1.0000
Pd1—Cl1	2.3045 (13)	C5—C6	1.415 (8)
Pd1—Cl1^i^	2.3045 (13)	C5—H5	1.0000
Fe1—C2	2.034 (5)	C6—H6	1.0000
Fe1—C10	2.036 (6)	C7—C11	1.415 (8)
Fe1—C6	2.040 (5)	C7—C8	1.427 (8)
Fe1—C9	2.041 (6)	C7—H7	1.0000
Fe1—C3	2.044 (5)	C8—C9	1.421 (8)
Fe1—C5	2.046 (5)	C8—H8	1.0000
Fe1—C7	2.046 (6)	C9—C10	1.434 (8)
Fe1—C11	2.050 (6)	C9—H9	1.0000
Fe1—C4	2.053 (6)	C10—C11	1.413 (9)
Fe1—C8	2.065 (5)	C10—H10	1.0000
O1—C13	1.369 (6)	C11—H11	1.0000
O1—C16	1.390 (7)	C12—C13	1.478 (8)
N1—C1	1.295 (6)	C12—H12A	0.9900
N1—C12	1.468 (7)	C12—H12B	0.9900
C1—C2	1.445 (7)	C13—C14	1.346 (8)
C1—H1	0.9500	C14—C15	1.419 (9)
C2—C3	1.430 (7)	C14—H14	0.9500
C2—C6	1.451 (7)	C15—C16	1.320 (9)
C3—C4	1.420 (7)	C15—H15	0.9500
C3—H3	1.0000	C16—H16	0.9500
			
N1—Pd1—N1^i^	180.0 (2)	C2—C3—H3	126.2
N1—Pd1—Cl1	90.75 (13)	Fe1—C3—H3	126.2
N1^i^—Pd1—Cl1	89.25 (13)	C5—C4—C3	108.7 (5)
N1—Pd1—Cl1^i^	89.25 (13)	C5—C4—Fe1	69.5 (3)
N1^i^—Pd1—Cl1^i^	90.75 (13)	C3—C4—Fe1	69.4 (3)
Cl1—Pd1—Cl1^i^	180.0	C5—C4—H4	125.6
C2—Fe1—C10	167.2 (2)	C3—C4—H4	125.6
C2—Fe1—C6	41.74 (19)	Fe1—C4—H4	125.6
C10—Fe1—C6	127.5 (2)	C6—C5—C4	108.6 (4)
C2—Fe1—C9	150.6 (2)	C6—C5—Fe1	69.5 (3)
C10—Fe1—C9	41.2 (2)	C4—C5—Fe1	70.0 (3)
C6—Fe1—C9	166.4 (2)	C6—C5—H5	125.7
C2—Fe1—C3	41.0 (2)	C4—C5—H5	125.7
C10—Fe1—C3	150.1 (2)	Fe1—C5—H5	125.7
C6—Fe1—C3	69.4 (2)	C5—C6—C2	107.3 (5)
C9—Fe1—C3	117.1 (2)	C5—C6—Fe1	70.0 (3)
C2—Fe1—C5	68.9 (2)	C2—C6—Fe1	68.9 (3)
C10—Fe1—C5	107.0 (2)	C5—C6—H6	126.3
C6—Fe1—C5	40.5 (2)	C2—C6—H6	126.3
C9—Fe1—C5	128.5 (2)	Fe1—C6—H6	126.3
C3—Fe1—C5	68.7 (2)	C11—C7—C8	108.6 (5)
C2—Fe1—C7	108.9 (2)	C11—C7—Fe1	70.0 (3)
C10—Fe1—C7	68.2 (2)	C8—C7—Fe1	70.4 (3)
C6—Fe1—C7	117.6 (2)	C11—C7—H7	125.7
C9—Fe1—C7	68.4 (2)	C8—C7—H7	125.7
C3—Fe1—C7	130.3 (2)	Fe1—C7—H7	125.7
C5—Fe1—C7	150.1 (2)	C9—C8—C7	107.4 (5)
C2—Fe1—C11	129.4 (2)	C9—C8—Fe1	68.8 (3)
C10—Fe1—C11	40.5 (3)	C7—C8—Fe1	68.9 (3)
C6—Fe1—C11	107.4 (2)	C9—C8—H8	126.3
C9—Fe1—C11	68.6 (2)	C7—C8—H8	126.3
C3—Fe1—C11	168.5 (2)	Fe1—C8—H8	126.3
C5—Fe1—C11	116.6 (2)	C8—C9—C10	107.9 (5)
C7—Fe1—C11	40.4 (2)	C8—C9—Fe1	70.7 (3)
C2—Fe1—C4	68.5 (2)	C10—C9—Fe1	69.2 (3)
C10—Fe1—C4	116.8 (2)	C8—C9—H9	126.1
C6—Fe1—C4	68.5 (2)	C10—C9—H9	126.1
C9—Fe1—C4	108.1 (2)	Fe1—C9—H9	126.1
C3—Fe1—C4	40.5 (2)	C11—C10—C9	108.0 (5)
C5—Fe1—C4	40.5 (2)	C11—C10—Fe1	70.3 (4)
C7—Fe1—C4	168.6 (2)	C9—C10—Fe1	69.6 (3)
C11—Fe1—C4	149.7 (2)	C11—C10—H10	126.0
C2—Fe1—C8	118.0 (2)	C9—C10—H10	126.0
C10—Fe1—C8	68.5 (2)	Fe1—C10—H10	126.0
C6—Fe1—C8	151.5 (2)	C10—C11—C7	108.1 (5)
C9—Fe1—C8	40.5 (2)	C10—C11—Fe1	69.2 (4)
C3—Fe1—C8	109.0 (2)	C7—C11—Fe1	69.6 (3)
C5—Fe1—C8	167.4 (2)	C10—C11—H11	126.0
C7—Fe1—C8	40.6 (2)	C7—C11—H11	126.0
C11—Fe1—C8	68.2 (2)	Fe1—C11—H11	126.0
C4—Fe1—C8	129.8 (2)	N1—C12—C13	111.9 (5)
C13—O1—C16	105.9 (5)	N1—C12—H12A	109.2
C1—N1—C12	116.9 (5)	C13—C12—H12A	109.2
C1—N1—Pd1	127.9 (4)	N1—C12—H12B	109.2
C12—N1—Pd1	115.1 (3)	C13—C12—H12B	109.2
N1—C1—C2	127.6 (5)	H12A—C12—H12B	107.9
N1—C1—H1	116.2	C14—C13—O1	109.9 (5)
C2—C1—H1	116.2	C14—C13—C12	134.6 (6)
C3—C2—C1	130.9 (5)	O1—C13—C12	115.5 (4)
C3—C2—C6	107.7 (5)	C13—C14—C15	106.6 (5)
C1—C2—C6	120.8 (5)	C13—C14—H14	126.7
C3—C2—Fe1	69.9 (3)	C15—C14—H14	126.7
C1—C2—Fe1	119.3 (4)	C16—C15—C14	107.5 (6)
C6—C2—Fe1	69.3 (3)	C16—C15—H15	126.2
C4—C3—C2	107.7 (4)	C14—C15—H15	126.2
C4—C3—Fe1	70.0 (3)	C15—C16—O1	110.0 (6)
C2—C3—Fe1	69.1 (3)	C15—C16—H16	125.0
C4—C3—H3	126.2	O1—C16—H16	125.0
			
Cl1—Pd1—N1—C1	75.8 (5)	C5—Fe1—C6—C2	−118.7 (5)
Cl1^i^—Pd1—N1—C1	−104.2 (5)	C7—Fe1—C6—C2	87.9 (4)
Cl1—Pd1—N1—C12	−106.5 (4)	C11—Fe1—C6—C2	130.4 (3)
Cl1^i^—Pd1—N1—C12	73.5 (4)	C4—Fe1—C6—C2	−81.4 (3)
C12—N1—C1—C2	178.8 (5)	C8—Fe1—C6—C2	54.2 (6)
Pd1—N1—C1—C2	−3.5 (8)	C2—Fe1—C7—C11	129.3 (4)
N1—C1—C2—C3	9.7 (10)	C10—Fe1—C7—C11	−37.4 (4)
N1—C1—C2—C6	179.8 (5)	C6—Fe1—C7—C11	84.6 (4)
N1—C1—C2—Fe1	97.6 (6)	C9—Fe1—C7—C11	−81.9 (4)
C10—Fe1—C2—C3	−154.9 (9)	C3—Fe1—C7—C11	170.3 (3)
C6—Fe1—C2—C3	−118.9 (4)	C5—Fe1—C7—C11	48.9 (6)
C9—Fe1—C2—C3	51.3 (6)	C4—Fe1—C7—C11	−155.9 (11)
C5—Fe1—C2—C3	−81.3 (3)	C8—Fe1—C7—C11	−119.3 (5)
C7—Fe1—C2—C3	130.5 (3)	C2—Fe1—C7—C8	−111.3 (4)
C11—Fe1—C2—C3	171.0 (3)	C10—Fe1—C7—C8	81.9 (4)
C4—Fe1—C2—C3	−37.7 (3)	C6—Fe1—C7—C8	−156.0 (3)
C8—Fe1—C2—C3	87.1 (3)	C9—Fe1—C7—C8	37.4 (3)
C10—Fe1—C2—C1	78.6 (11)	C3—Fe1—C7—C8	−70.4 (4)
C6—Fe1—C2—C1	114.6 (6)	C5—Fe1—C7—C8	168.3 (4)
C9—Fe1—C2—C1	−75.2 (7)	C11—Fe1—C7—C8	119.3 (5)
C3—Fe1—C2—C1	−126.5 (5)	C4—Fe1—C7—C8	−36.5 (14)
C5—Fe1—C2—C1	152.2 (5)	C11—C7—C8—C9	1.6 (7)
C7—Fe1—C2—C1	4.0 (5)	Fe1—C7—C8—C9	−58.2 (4)
C11—Fe1—C2—C1	44.5 (5)	C11—C7—C8—Fe1	59.8 (4)
C4—Fe1—C2—C1	−164.1 (5)	C2—Fe1—C8—C9	−153.7 (3)
C8—Fe1—C2—C1	−39.4 (5)	C10—Fe1—C8—C9	38.4 (4)
C10—Fe1—C2—C6	−36.0 (11)	C6—Fe1—C8—C9	168.5 (4)
C9—Fe1—C2—C6	170.2 (4)	C3—Fe1—C8—C9	−109.8 (4)
C3—Fe1—C2—C6	118.9 (4)	C5—Fe1—C8—C9	−32.8 (12)
C5—Fe1—C2—C6	37.6 (3)	C7—Fe1—C8—C9	119.6 (5)
C7—Fe1—C2—C6	−110.6 (4)	C11—Fe1—C8—C9	82.1 (4)
C11—Fe1—C2—C6	−70.1 (4)	C4—Fe1—C8—C9	−69.3 (4)
C4—Fe1—C2—C6	81.3 (3)	C2—Fe1—C8—C7	86.7 (4)
C8—Fe1—C2—C6	−154.0 (3)	C10—Fe1—C8—C7	−81.2 (4)
C1—C2—C3—C4	171.5 (5)	C6—Fe1—C8—C7	49.0 (6)
C6—C2—C3—C4	0.4 (6)	C9—Fe1—C8—C7	−119.6 (5)
Fe1—C2—C3—C4	59.6 (4)	C3—Fe1—C8—C7	130.6 (3)
C1—C2—C3—Fe1	111.8 (6)	C5—Fe1—C8—C7	−152.4 (9)
C6—C2—C3—Fe1	−59.3 (4)	C11—Fe1—C8—C7	−37.5 (4)
C2—Fe1—C3—C4	−119.0 (4)	C4—Fe1—C8—C7	171.2 (3)
C10—Fe1—C3—C4	50.1 (5)	C7—C8—C9—C10	−1.2 (6)
C6—Fe1—C3—C4	−80.5 (3)	Fe1—C8—C9—C10	−59.5 (4)
C9—Fe1—C3—C4	86.5 (4)	C7—C8—C9—Fe1	58.3 (4)
C5—Fe1—C3—C4	−37.0 (3)	C2—Fe1—C9—C8	52.8 (6)
C7—Fe1—C3—C4	170.2 (3)	C10—Fe1—C9—C8	−118.7 (5)
C11—Fe1—C3—C4	−156.4 (10)	C6—Fe1—C9—C8	−156.1 (9)
C8—Fe1—C3—C4	129.8 (3)	C3—Fe1—C9—C8	87.9 (4)
C10—Fe1—C3—C2	169.1 (4)	C5—Fe1—C9—C8	171.3 (3)
C6—Fe1—C3—C2	38.5 (3)	C7—Fe1—C9—C8	−37.5 (4)
C9—Fe1—C3—C2	−154.5 (3)	C11—Fe1—C9—C8	−81.1 (4)
C5—Fe1—C3—C2	82.0 (3)	C4—Fe1—C9—C8	130.9 (3)
C7—Fe1—C3—C2	−70.8 (4)	C2—Fe1—C9—C10	171.5 (4)
C11—Fe1—C3—C2	−37.4 (11)	C6—Fe1—C9—C10	−37.4 (12)
C4—Fe1—C3—C2	119.0 (4)	C3—Fe1—C9—C10	−153.4 (3)
C8—Fe1—C3—C2	−111.2 (3)	C5—Fe1—C9—C10	−70.0 (4)
C2—C3—C4—C5	−0.6 (6)	C7—Fe1—C9—C10	81.2 (4)
Fe1—C3—C4—C5	58.5 (4)	C11—Fe1—C9—C10	37.6 (4)
C2—C3—C4—Fe1	−59.0 (4)	C4—Fe1—C9—C10	−110.4 (4)
C2—Fe1—C4—C5	−82.3 (3)	C8—Fe1—C9—C10	118.7 (5)
C10—Fe1—C4—C5	84.9 (4)	C8—C9—C10—C11	0.4 (7)
C6—Fe1—C4—C5	−37.3 (3)	Fe1—C9—C10—C11	−60.0 (4)
C9—Fe1—C4—C5	128.7 (3)	C8—C9—C10—Fe1	60.4 (4)
C3—Fe1—C4—C5	−120.4 (5)	C2—Fe1—C10—C11	−41.9 (11)
C7—Fe1—C4—C5	−161.3 (11)	C6—Fe1—C10—C11	−71.4 (4)
C11—Fe1—C4—C5	50.5 (6)	C9—Fe1—C10—C11	118.9 (5)
C8—Fe1—C4—C5	168.5 (3)	C3—Fe1—C10—C11	172.1 (4)
C2—Fe1—C4—C3	38.1 (3)	C5—Fe1—C10—C11	−111.3 (4)
C10—Fe1—C4—C3	−154.6 (3)	C7—Fe1—C10—C11	37.4 (3)
C6—Fe1—C4—C3	83.1 (3)	C4—Fe1—C10—C11	−153.9 (3)
C9—Fe1—C4—C3	−110.8 (3)	C8—Fe1—C10—C11	81.2 (4)
C5—Fe1—C4—C3	120.4 (5)	C2—Fe1—C10—C9	−160.8 (9)
C7—Fe1—C4—C3	−40.8 (13)	C6—Fe1—C10—C9	169.6 (3)
C11—Fe1—C4—C3	170.9 (4)	C3—Fe1—C10—C9	53.2 (6)
C8—Fe1—C4—C3	−71.1 (4)	C5—Fe1—C10—C9	129.8 (4)
C3—C4—C5—C6	0.5 (6)	C7—Fe1—C10—C9	−81.6 (4)
Fe1—C4—C5—C6	59.0 (4)	C11—Fe1—C10—C9	−118.9 (5)
C3—C4—C5—Fe1	−58.5 (4)	C4—Fe1—C10—C9	87.2 (4)
C2—Fe1—C5—C6	−38.7 (3)	C8—Fe1—C10—C9	−37.7 (4)
C10—Fe1—C5—C6	128.5 (3)	C9—C10—C11—C7	0.6 (7)
C9—Fe1—C5—C6	168.8 (3)	Fe1—C10—C11—C7	−59.0 (4)
C3—Fe1—C5—C6	−82.9 (3)	C9—C10—C11—Fe1	59.6 (4)
C7—Fe1—C5—C6	52.8 (6)	C8—C7—C11—C10	−1.3 (7)
C11—Fe1—C5—C6	85.9 (4)	Fe1—C7—C11—C10	58.7 (4)
C4—Fe1—C5—C6	−119.9 (4)	C8—C7—C11—Fe1	−60.1 (4)
C8—Fe1—C5—C6	−164.5 (9)	C2—Fe1—C11—C10	169.0 (3)
C2—Fe1—C5—C4	81.2 (3)	C6—Fe1—C11—C10	128.0 (3)
C10—Fe1—C5—C4	−111.7 (4)	C9—Fe1—C11—C10	−38.3 (3)
C6—Fe1—C5—C4	119.9 (4)	C3—Fe1—C11—C10	−159.9 (9)
C9—Fe1—C5—C4	−71.3 (4)	C5—Fe1—C11—C10	85.2 (4)
C3—Fe1—C5—C4	37.0 (3)	C7—Fe1—C11—C10	−119.6 (5)
C7—Fe1—C5—C4	172.7 (4)	C4—Fe1—C11—C10	51.1 (6)
C11—Fe1—C5—C4	−154.2 (3)	C8—Fe1—C11—C10	−82.0 (4)
C8—Fe1—C5—C4	−44.6 (11)	C2—Fe1—C11—C7	−71.4 (4)
C4—C5—C6—C2	−0.3 (6)	C10—Fe1—C11—C7	119.6 (5)
Fe1—C5—C6—C2	59.0 (4)	C6—Fe1—C11—C7	−112.4 (4)
C4—C5—C6—Fe1	−59.3 (4)	C9—Fe1—C11—C7	81.4 (4)
C3—C2—C6—C5	−0.1 (6)	C3—Fe1—C11—C7	−40.3 (12)
C1—C2—C6—C5	−172.2 (5)	C5—Fe1—C11—C7	−155.2 (4)
Fe1—C2—C6—C5	−59.7 (4)	C4—Fe1—C11—C7	170.8 (4)
C3—C2—C6—Fe1	59.6 (4)	C8—Fe1—C11—C7	37.7 (4)
C1—C2—C6—Fe1	−112.6 (5)	C1—N1—C12—C13	−105.6 (6)
C2—Fe1—C6—C5	118.7 (5)	Pd1—N1—C12—C13	76.5 (5)
C10—Fe1—C6—C5	−70.7 (4)	C16—O1—C13—C14	1.4 (7)
C9—Fe1—C6—C5	−40.4 (11)	C16—O1—C13—C12	−179.1 (5)
C3—Fe1—C6—C5	80.9 (3)	N1—C12—C13—C14	−117.7 (7)
C7—Fe1—C6—C5	−153.4 (3)	N1—C12—C13—O1	62.9 (7)
C11—Fe1—C6—C5	−110.8 (3)	O1—C13—C14—C15	−1.0 (7)
C4—Fe1—C6—C5	37.3 (3)	C12—C13—C14—C15	179.6 (7)
C8—Fe1—C6—C5	173.0 (4)	C13—C14—C15—C16	0.2 (8)
C10—Fe1—C6—C2	170.6 (3)	C14—C15—C16—O1	0.7 (9)
C9—Fe1—C6—C2	−159.1 (9)	C13—O1—C16—C15	−1.3 (8)
C3—Fe1—C6—C2	−37.9 (3)		

Symmetry code: (i) −*x*+1, −*y*, −*z*+1.
